# Oil Sorption and Oil–Water Separation Using 3D Printed Sorbents Manufactured with Fused Filament Fabrication

**DOI:** 10.3390/polym18172138

**Published:** 2026-09-02

**Authors:** József Kántor, Attila-Levente Gergely

**Affiliations:** Department of Mechanical Engineering, Faculty of Technical and Human Sciences Târgu Mureş, Sapientia Hungarian University of Transylvania, Corunca, Calea Sighișoarei nr. 2., 540485 Târgu Mureş, Romania; kantorjozsef@ms.sapientia.ro

**Keywords:** sorbents, FFF 3D printing, oil sorption, fibrous structures, reusability

## Abstract

Fiber-based sorbents are widely used and researched for oil spill treatment. The sorption capacity of natural and synthetic fibers ranges from poor to exceptional; however, the preparation of such fiber-based sorbents requires specialized equipment, and the reusability is still unresolved. This paper presents sorbents prepared for the first time by Fused Filament Fabrication 3D printing. The sorbents contain capillaries ranging from 1 to 6 mm that were created by depositing the filament in grid and rectilinear infill patterns. Sorption kinetics and maximum capillary rise were investigated for diesel, and 5W30 and 85W140 oils in the cases of PP, rPET, and PLA as sorbent materials. Capillary size influences sorption capacity and sorption kinetics, 0.7 g/g for 1 mm and 2.7 g/g for 4 mm, whereas at 10 s the relative capillary rise was 64.1% for 1 mm and 86.8% for 4 mm. Viscosity has a significant effect on the sorption kinetics. A special sorbent design is proposed to increase the sorption capacity to 5.26 g/g. Reusability was tested and the sorption capacity stayed constant over 10 cycles. Although the sorption capacity is lower compared to electrospun nanofibers, the oil release does not require any specialty equipment and it takes 7–8 s, making this type of sorbent highly desirable in practical applications.

## 1. Introduction

Energy availability is essential for humanity to live, to produce, and to innovate in technologies that further increase the standard of living. As countries become more developed, the energy usage per capita increases due to the higher standard of living [1]. Based on energy usage data, the International Energy Agency showed that energy consumption per capita in the world increased by 16% between 2000 and 2023 [2]. While the energy mix of each country is uniquely composed based on available natural resources, geographic location, neighboring countries, available infrastructure, etc., in general, oil-based energy carriers play an important part. Oil products make up a significant part, ~38%, of the energy mix, of the European countries combined, whereas renewables account for less than 20% [3]. Oil and oil products constitute ~30% of the energy mix of the world [2]. Inevitably, handling more oil and oil products leads to more accidents or deliberate discharges, contaminating terrestrial and marine environments [4,5]. Oil spills have devastating effects on the marine environment; moreover, spills in fresh water such as lakes, rivers, streams and wetlands can cause the pollution of drinking water [6].

There are different ways to clean up spilled oil and oil products from water surfaces: (1) the use of chemicals that break down the oil, (2) mechanical means that can remove the oil from the water surface, (3) in situ burning, or (4) bioremediation [7]. While all the methods have advantages and disadvantages, the mechanical approach provides a timely and effective response without further contaminating the spill site with chemicals or the byproducts of the burnt oil. Depending on the severity of the oil spill, the mechanical approach provides different solutions: for larger spills, booms and skimmers are used to contain and collect the oil; for smaller spills, sorbents can be used. Sorbents are porous structures that collect and retain oil, thus the oil can be transferred from the water surface to containers [8].

The use of sorbents is the most environmentally friendly solution to remove oil films from water surfaces [9,10]. Natural materials such as lignin, wood waste, cotton, hemp, wool, paper, etc., have been investigated for potential sorbent materials. However, in spite of their low cost, there are several shortcomings that make these materials unfit to be used as sorbents, such as low sorption capacity (3–8 g/g), limited or inability for repeated reuse [11]. On the other hand, synthetic polymeric materials have shown significantly better sorption capacity and reusability [12]. Polymeric fibrous structures have high surface-to-volume ratios, and the chemical structure of the polymeric material can be chosen in such a way that it facilitates the sorption of a particular oil or oil derivative. There are multiple studies that deal with the development of polymeric sorbents. Polystyrene (PS) has been shown to have a sorption capacity of over 50 g/g [13], whereas polylactic acid (PLA), depending on the fiber diameters, showed 25–30 g/g (d = 4 μm) [14], or 95 g/g (d = 0.82 ± 0.15 μm) [15]; polypropylene (PP) fibers (d = 4–7 μm) showed a sorption capacity of 80–129 g/g [16].

The preparation of polymeric fibrous structures can be achieved by several techniques, of which melt blowing is the established industrial method. Nevertheless, it is rarely used in laboratory scale applications because it requires high upfront capital investment; furthermore, it is difficult to control the fiber size [17]. The scientific community readily uses solution or melt electrospinning and centrifugal spinning to prepare fibers on the nanometer scale [18,19]. Electrospinning, in general, has a low production rate, 0.1 g/h, whereas in the case of centrifugal spinning, while having a higher production rate, the produced fiber diameters are in the micrometer range [20]. Fibrous structures prepared by electrospinning or centrifugal spinning have high potential in sorbent applications, however the reusability of such sorbents requires specialized equipment. Methods, such as compression and centrifuging, have been used to extract the oil from sorbents. These methods require either a press or a centrifuge that would be difficult to operate at the spill site.

In the past couple of years, Material Extrusion, more particularly, Fused Filament Fabrication (FFF) 3D printing, has undergone significant development. This technology is capable of producing plastic parts with high precision and complexity. It is estimated that for under ~50,000 units, it is more beneficial to produce with FFF 3D printing than with injection molding [21]. 3D printing has several processing parameters that influence the outcome, and the infill parameter is a simple way of creating fibrous sorbents with different porosities. A further benefit of FFF 3D printing is the wide range of printable geometries; thus, using 3D printing to produce fibrous polymeric structures is most appealing. 3D printed structures can be created using various materials, so the sorbent material can be tuned to the contaminant. Based on our research, there is a limited number of studies available dealing with oil sorption using 3D printed sorbents. Lichade et al. used video-projection-based stereolithography 3D printing to prepare sorbents with gradient porous structures to separate and filter oil from water [22]. Although the separation experiments were successful, the reusability of the sorbents was not discussed. Furthermore, video-projection-based stereolithography 3D printing is more expensive, and the raw materials are more limited than in the case of FFF 3D printing. 3D printed porous structures have been used for catalysis and dye filtration applications [23,24,25]. Li et al. used direct-ink-writing 3D printing to prepare sorbents of porous carbon and foaming gelatin aerogel. The authors found that the prepared sorbents were ~95% effective in absorbing anionic and cationic dyes from water [26]. In a recent publication, Mercado-Rico et al. reported the development of 3D printable sodium alginate–bovine serum albumin porous cryogels [27]. Different compositions of the material showed oil sorption capacities of 1.5–4 g/g, but they did not test the 3D printed structures themselves. In conclusion, 3D printing with the technological advances that happened in the last couple of years is a promising technology that can be used to create porous fiber-based sorbents for oil–water separation.

The aim of this study is to prepare fibrous polymeric sorbents suitable for oil sorption and oil–water separation applications with FFF 3D printing. Based on our literature search, this is the first report to address the use of FFF 3D printing in such an application. Furthermore, we examine the capillary effect of 3D printed sorbents, test different sorbent materials with various oils, and investigate the kinetics of the sorption process. A sorbent structure is designed for increased porosity and sorption capacity, as well as rapid reusability.

## 2. Materials and Methods

### 2.1. Materials

Polylactic acid (PLA, 1.75 mm, Prusament PLA Transparent, Prusa Research a.s., Prague, Czech Republic) and recycled polyethylene terephthalate (rPET, 1.75 mm, BASF Ultrafuse rPET natural blue, Mass Additive Manufacturing GmbH, Heidelberg, Germany) filaments were dried for 6 h at 55 °C before use, and polypropylene (PP, 1.75 mm, BASF Ultrafuse PP natural, Mass Additive Manufacturing GmbH, Heidelberg, Germany) filament was used as received. 5W30 motor oil (Castrol Edge, Pangbourne, UK), 85W140 mineral transmission gear oil (Kross Trans GL-5), and standard diesel fuel (MOL Romania Petroleum Products SRL, Cluj-Napoca, Romania) were used as received. Polypropylene sorbent padding (RS PRO Pad Spill Absorbent, RS Components Ltd., Bucharest, Romania) was used without any modification. Tap water was used in the water contact angle measurements and the oil–water separation experiments.

### 2.2. Modeling

The capillary tubes were modeled in Blender (5.1.2, Blender Foundation, Amsterdam, The Netherlands) and printed as outer walls, without any top/bottom layers and infill. There were grid and rectilinear structures as well. The 1 and 1.5 mm capillaries were printed in a 2 × 2 configuration for the sake of printability, while the larger capillaries were a singular tube.

### 2.3. Fused Filament Fabrication (FFF) 3D Printing

FFF 3D printing was performed with a Sovol SV07 printer (Shenzhen Lian Dian Chuang Technology Co. Ltd., Shenzhen, China). All specimens were printed with 0.45 mm line width and 0.2 mm layer thickness. PLA specimens were printed at 215 °C nozzle temperature, 60 °C bed temperature and 50 mm/s printing speed. For rPET these values were 235 °C, 75 °C, and 50 mm/s, respectively, and for PP 240 °C, 80 °C, and 40 mm/s, respectively. The speed of the auxiliary cooling fan was kept at 70% in all cases.

For slicing, OrcaSlicer (open-source, ver. 2.3.1.) was used. The infill-based specimens were printed without walls and top/bottom layers, and had varying infills according to the capillary size. The 1, 1.5, 2, 3, 4, 5, and 6 mm capillary sizes respectively corresponded to 28%, 20.9%, 16.6%, 11.8%, 9.1%, 7.5% and 6.3% infills with rectilinear patterns, and double of that for the grid infill.

### 2.4. Surface Tension

The measurement of surface tension was carried out using pendent drop tensiometry based on the research of Berry et al. [28]. Photographs of pendent drops were taken with a Canon EOS R50 digital camera (Canon Inc., Tokyo, Japan) equipped with a Venus Optics Laowa 100 mm macro lens (Anhui ChangGeng Optics Technology Co., Ltd., Hefei, China). The images were analyzed in OpenDrop 3.3.2. All measurements were repeated 3 times.

### 2.5. Viscosity Measurement

The dynamic viscosity of the oils was measured with an IKA ROTAVISC lo-vi viscometer (IKA, Königswinter, Germany) at 22.5 ± 0.5 °C. Depending on the oil, different shear rates were used to stay within the range of the equipment. Based on the dimensions of the spindle and the oil container, the chosen 40 and 100 rpm rotational speeds resulted in shear rates of 2.89 and 7.25 1/s, respectively. The viscosity values were recorded 1 min after starting the rotation of the spindle.

### 2.6. Contact Angle (CA) Measurement

The contact angle measurements were performed using a Canon EOS R50 digital camera (Canon Inc., Tokyo, Japan) equipped with a Venus Optics Laowa 100 mm macro lens (Anhui ChangGeng Optics Technology Co., Ltd., Hefei, China). The images were analyzed in OpenDrop 3.3.2. The measurements were carried out on flat surfaces prepared from the filament materials by compression molding. Photographs were taken 5 s after placing the droplets onto the surface. The average value was calculated based on 6 measurements.

### 2.7. Microscopy

Optical microscopy was used to study the 3D printed structures. The investigation was performed with a Carl Zeiss toolmaker’s microscope (Carl Zeiss AG, Oberkochen, Germany) equipped with a digital camera.

### 2.8. Porosity Calculation

The dimensions of the rectangular prismatic specimens were measured with a digital caliper. The density of the sorbents was calculated by dividing the mass of the mat with its volume:

(1) $$\rho_{sorb}=\frac{m_{sorb}}{V_{mat}}$$

From here, the porosity could be calculated as follows:

(2) $$porosity=1-\frac{\rho_{sorb}}{\rho_{bulk}}$$

where the *ρ*_bulk_ is the density of the bulk sorbent material.

### 2.9. Oil Sorption Studies

To determine the oils sorption properties, and the capability and capacity of the 3D printed sorbents, several experiments were performed that can be divided into the following categories. The experiments were performed under standard laboratory conditions (22 °C, 50% RH).

#### 2.9.1. Capillary Rise and Kinetics Study

The 3D printed rectilinear and grid capillary tubes were dipped into an oil reservoir, and video was recorded as the oil level rose. Three sets of experiments were performed in this configuration:

1. PLA capillaries of 1, 1.5, 2, 3, 4 mm and 5W30 motor oil;
2. PLA, rPET, and PP capillaries of 1.5 mm and 5W30 motor oil;
3. PLA capillaries of 1 mm with diesel, 5W30 motor oil, and 85W140 transmission oil.

From the image data, the maximum capillary rise as well as capillary rise over time were acquired. The experiments were carried out 3 times.

#### 2.9.2. Oil Sorption Capacity Measurements

Rectilinear infill-based PLA specimens of 1, 1.5, 2, and 3 mm capillary sizes and 1, 2, 3, 4, 5, 6, 7, 8, 9, and 10 mm thicknesses were exposed to 5W30 motor oil for 30 s, during which the specimens were fully saturated with oil. Upon removal, the specimens were drained for 30 s and weighed. The experiments were carried out 3 times.

#### 2.9.3. Oil–Water Separation Study

The oil–water separation experiments were performed by adding 1.2 g of 5W30 motor oil on the surface of 50 mL of water. Rectilinear infill-based PLA, rPET, and PP specimens were held with a pair of tweezers and dipped into the liquids for 30 s. After that, the specimens were removed, drained for 30 s, and weighed. The leftover oil in the beaker was collected with melt-blown PP fiber mat specimens to precisely determine the absorbed oil quantity. The oil uptake by the PP fiber mat specimens was determined with the help of a calibration curve. The experiments were carried out 3 times.

#### 2.9.4. Reusability Study

The reusability study was carried out with a specially designed rectilinear specimen with high porosity. The specimen was placed in the oil for 10 s, after which it was lifted and drained for 30 s. The oil removal was performed by placing the sorbent onto a porous platform. The sorption–release cycle was repeated 10 times.

## 3. Results and Discussion

### 3.1. Printed Sorbent Characterization

Highly porous structures were created with FFF 3D printing with the intention to study the oil sorption mechanism. Using the analogy with nano- and microfibrous mats, the 3D printed structures had a fiber size on the order of 100 s of µm, with pore sizes ranging from 1 to 6 mm; their surface areas were lower by 2–3 orders of magnitude, and the fiber alignment was highly organized. In this study, three materials were tested: PLA, rPET and PP. PLA, being the most commonly used material in FFF 3D printing, served as a model for the printing process, with the added benefit that it is a biomaterial and its precursors can be derived from biomass. Recycled PET is an attractive option because it is among the most common polymers produced, and, as such, there is a high demand for recycling. PP is commonly used in commercial oil sorbents as melt-blown fibers, and it was used as the control. In general, fiber-based sorbents should have the following characteristics: (1) the sorbent material should have certain affinity to the absorbed liquid and preferably derived from a sustainable resource; (2) the sorbent should be inexpensive and (3) mechanically robust; and (4) it should have good sorption capacity and (5) fast sorption and release capability, promoting reusability without specialized equipment.

To investigate the sorption process of the liquids, certain physical properties are required. Table 1 presents the surface tension (γ), density (*ρ*), and dynamic viscosity (η) of the studied motor oil (5W30), gear oil (85W140), and diesel.

Water contact angle (WCA) and oil contact angle (OCA) measurements were performed on the polymeric materials, in order to determine the wettability of the studied polymeric surfaces by the different oils (Table 2). The WCA was the smallest for PLA, whereas highest for PP. Our results correlate well with WCA data reported in the literature [29,30].

**Figure 1:**
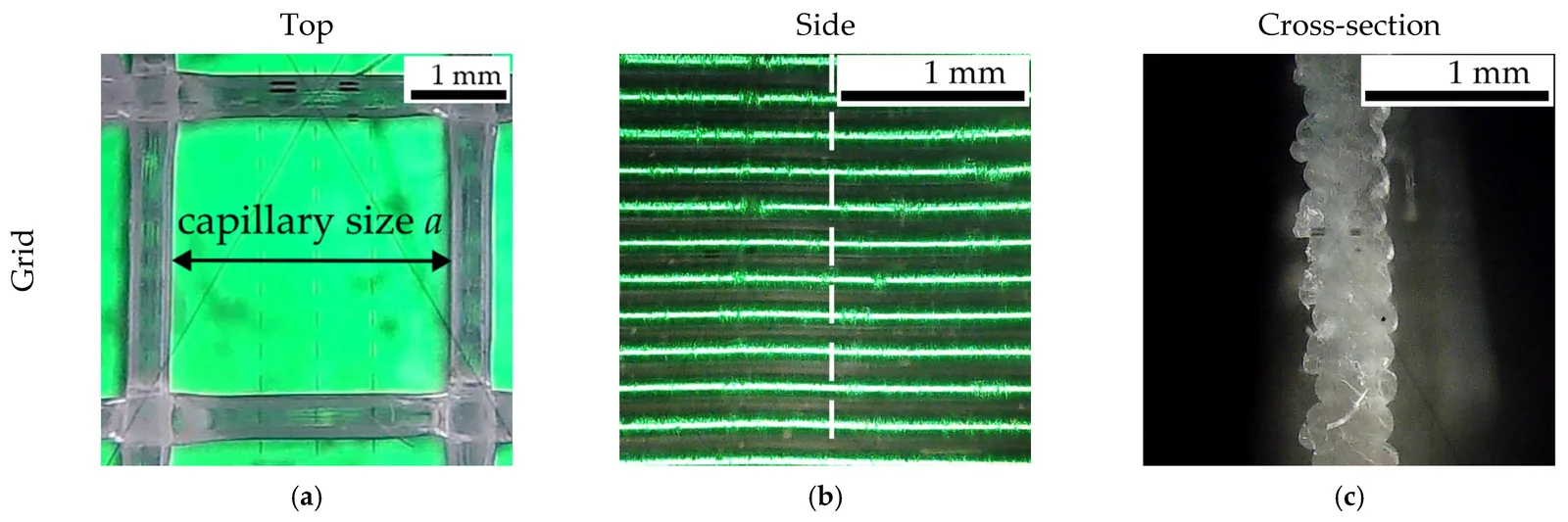

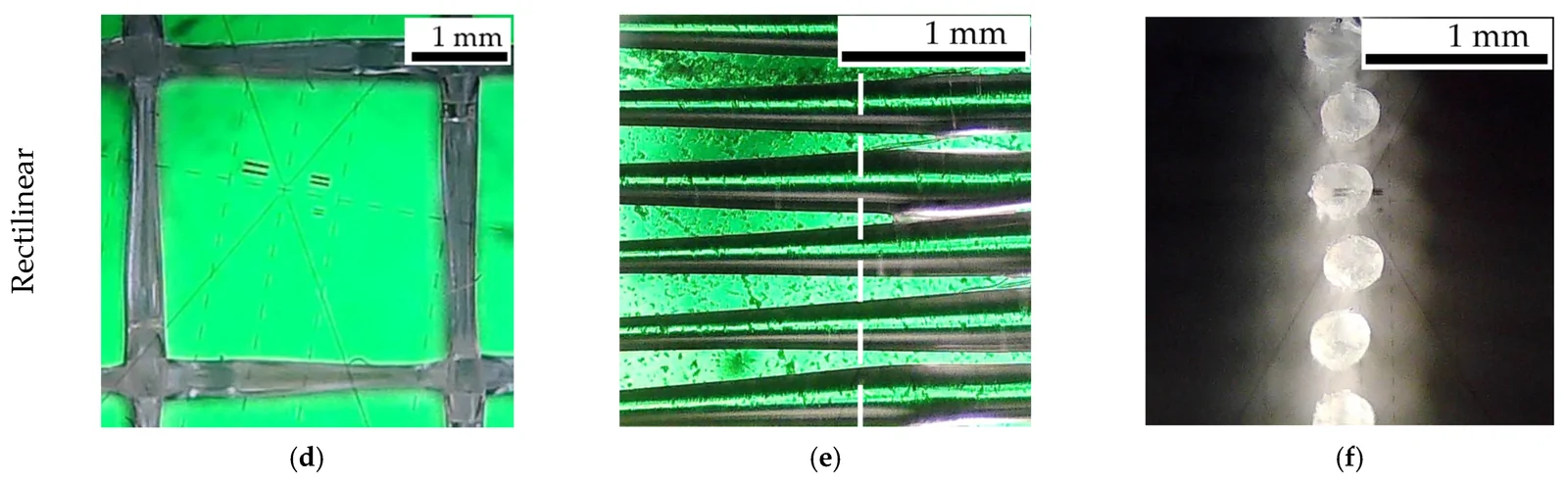
Micrographs of grid and rectilinear PLA specimens: (**a**,**d**) top, (**b**,**e**) side, and (**c**,**f**) cross-section views. The dashed lines in (**b**,**e**) represent the projection of the plane along which the cross-section was created.

The porous structures that were prepared by 3D printing contain a set of channels that can be treated as capillaries, which are essential for sorption. Representative 3D printed samples are shown in Figure 1 in grid and rectilinear configurations. Grid structure is created by depositing perpendicular lines in every layer, whereas the rectilinear structure is created by parallel lines, the direction of which alternates by 90° in each layer. This means that in the rectilinear pattern, only 50% material is needed when compared to grid. The theoretical layer height during printing was set to 0.2 mm and the wall thickness (line width in OrcaSlicer) to 0.45 mm. Measurements showed that the printing process resulted in sorbents with 0.2 mm layer height for both samples, matching the theoretical value. In the case of grid samples, a continuous wall with a certain surface roughness and 0.45 ± 0.02 mm wall thickness was created (Figure 1c). In contrast, the rectilinear configuration had non-continuous walls with clearly visible gaps. The fibers had a conical shape between two walls (Figure 1e), with the size ranging from 0.25 to 0.4 mm. This is due to the fact that the extruded layer during the printing process is unsupported, therefore the individual layers retain their circular cross-section, as can be seen in Figure 1f.

Table 3 shows the theoretical and measured capillary size (a, Figure 1a) for samples with grid and rectilinear configuration. The capillaries had square cross-sections, as shown in Figure 1a,d, with their sides being the characteristic length. Samples with different capillary sizes were printed. The measured capillary size of the grid samples showed lower values compared to the theoretical one, and as the capillary size increased, the measured value got closer to the theoretical. This is due to the fact that every time the 3D printer’s nozzle passes through a node, additional material is being deposited on the node. Since the nozzle position on the Z axis does not change, the extra material is dispersed along the wall. The node density is inversely proportional to the capillary size, thus the smaller the capillary size, the higher the deviation of the measured value compared to the theoretical value. In the case of rectilinear structure, the measured values were within 5% of the theoretical value.

Designing the 3D printed sorbent structure in any 3D modeling software requires a lot of work and/or computing power (if parametric modeling is used). To simplify this process, one can use the infill function of the slicer, and thus a sorbent structure with the desired porosity can be prepared. To study the dependability of the infill function of the slicer on the porosity of the sorbents, the base area of the sorbents and the capillary size were studied. In theory, the infill value of a 3D printed object that has no walls and top/bottom layers directly correlates to the porosity. In order to produce structures with the same capillary size, grid infill has to be exactly double of the rectilinear infill. For instance, a grid of a = 1.5 mm capillaries in rectilinear configuration has an infill of 20.9% and, thus, a theoretical porosity of 79.1%, while in grid configuration the values are 41.8% and 58.2%, respectively.

**Figure 2:**
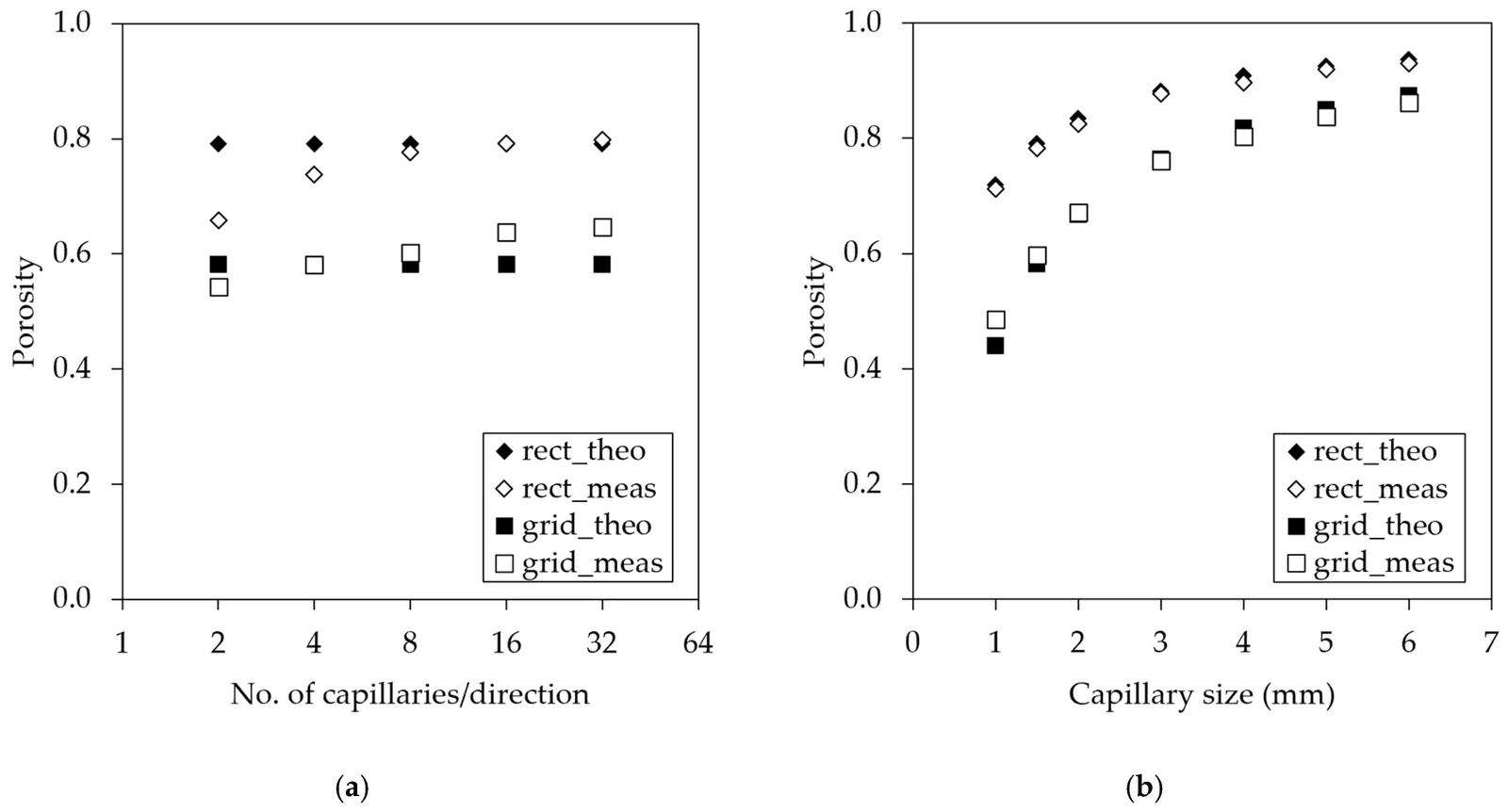
(**a**) Theoretical and measured porosities as a function of number of capillaries for a = 1.5 mm and (**b**) porosities as a function of capillary size.

Because of the way the toolpath is created, the outer walls of a rectilinear infill pattern contain some additional material where the nozzle makes a 180° direction change, resulting in continuous outer wall segments. Therefore, the porosity of the printed sorbent is influenced by this phenomenon at a greater degree when the sample’s base area is small. Figure 2a shows the theoretical and measured porosity values of rectilinear and grid structures of a = 1.5 mm as the number of capillaries increases from 2 × 2 to 32 × 32. The grid samples start out close to the theoretical and eventually overshoot, while the rectilinear ones show significantly lower porosities of 65.8% at 2 × 2 and 73.7% at 4 × 4, respectively, with the difference disappearing as samples get larger and the circumference-to-area ratio decreases. The datapoints in Figure 2 contain the error bars; however, the error is so low that it cannot be seen.

In Figure 2b, the change of porosity is shown as a function of capillary size. While in theory the porosity can be increased to nearly 100%, it does not provide any real value after a certain size as the capillaries become too large to hold any liquid. In the figure, the highest value shown is 93.7% for rectilinear and 87.4% for grid at 6 mm capillary size. In conclusion, the measured values show a good agreement with the ones calculated from the infill, indicating that the infill parameter of FFF 3D printing provides excellent control over the porosity of the specimens when the sorbents contain more than 4 × 4 capillaries and the capillary size is higher than 1.5 mm.

### 3.2. Capillary Rise and Kinetics

To examine the sorption capacity of the FFF 3D printed porous specimens, the capillary effect has to be investigated for the square cross-section. The sorption capacity is directly connected to the capillary rise, which for a given cross section can be calculated with the help of the capillary pressure, described by the Young–Laplace equation. When the oil comes into contact with the capillary wall, the oil surface becomes curved, forming a meniscus, and a pressure difference is observed between the two sides of the meniscus, as follows:

(3) $$∆p=\gamma \left(\frac{1}{R_{1}}+\frac{1}{R_{2}}\right)=\frac{2\gamma }{R}=\frac{2\gamma \cos\theta }{r}$$

where γ is the surface tension, R is the radius of curvature of the meniscus in a circular capillary, r is the radius of the capillary, and θ is the CA. Because there is a lower pressure zone under the meniscus, the atmospheric pressure on the liquid body causes the liquid to rise in the capillary until the hydrostatic pressure of the liquid column becomes equal to the pressure difference. Substituting Δp with the hydrostatic pressure and rearranging the equation gives the equilibrium height, which is known as Jurin’s law [31]:

(4) $$h=\frac{2\gamma \cos\theta }{∆\rho gr}$$

where Δ*ρ* is the density difference between the two media and g is the gravitational acceleration. There are several models that adapt Jurin’s law to non-circular capillaries. Wu et al. published a general expression for square capillaries that takes the non-zero CA into consideration [32]. The equilibrium height for square capillaries is as follows:

(5) $$h=\frac{2\gamma (\cos\theta +\sqrt{\cos\theta \sin\theta +\theta -\frac{\pi }{4}})}{∆\rho ga}$$

where a is the capillary size (side length of the square cross-section).

**Figure 3:**
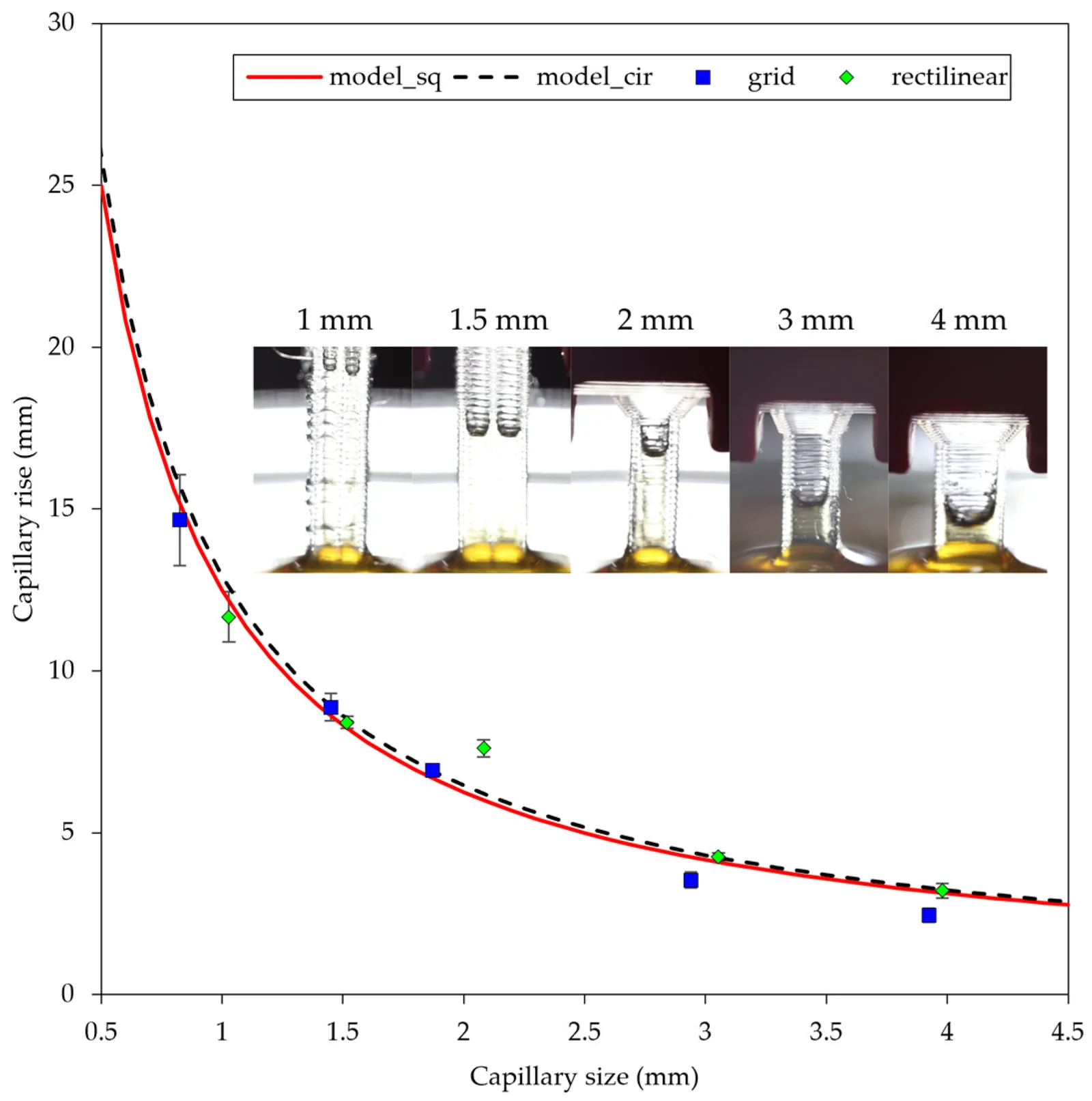
Maximum capillary rise as a function of capillary size for PLA and 5W30 oil. Representative capillary rise images are illustrated for the rectilinear configuration for a = 1, 1.5, 2, 3, and 4 mm.

In the first test, the maximum capillary rise was measured with PLA capillaries and motor oil. Figure 3 shows the equilibrium height for square capillaries based on Equation (5) and circular capillaries (calculated with Equation (4)), as well as the experimental data acquired for various capillary sizes. The input constants for the theoretical calculations were γ = 31.48 ± 0.05 mN/m *ρ* = 0.860 g/cm^3^, OCA = 30°. While the capillary rise was measured from the bottom of the meniscus, the oil level reached much higher in the corners, as published earlier [33]. The data points follow the curve generated with the model, indicating that the square capillary approximation provides a good description for the printed specimens. The biggest difference was in cases of the 3 and 4 mm grid specimens, where the maximum rise results were 17.8% and 23.4% lower than predicted, respectively. This difference at higher capillary sizes could be due to measurement error, because the position of the meniscus was more difficult to determine on the obtained images. The measured capillary rise of the rectilinear specimens was in good agreement with the theory. The difference between the square and circular capillary models is smaller than the experimental error, indicating that the printed capillaries can be treated as being circular, with the side length corresponding to the circle diameter.

**Figure 4:**
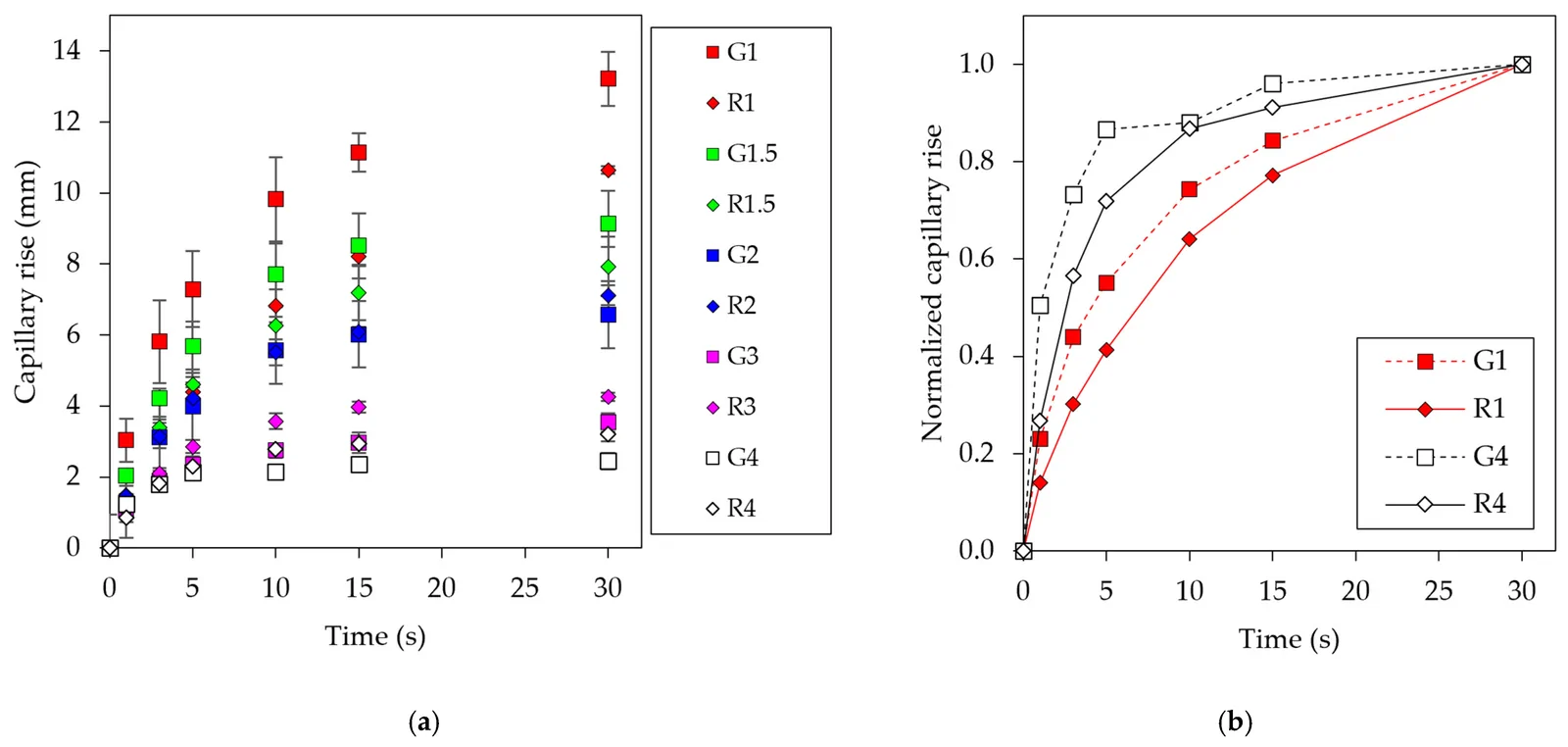
Sorption kinetics for PLA and 5W30 motor oil. (**a**) Capillary sizes of 1, 1.5, 2, 3 and 4 mm in grid and rectilinear structures and (**b**) normalized capillary rise. R—rectilinear and G—grid configuration.

While the maximum capillary rise is dictated by thermodynamics, in the same experiment, the kinetics of capillary action can also be measured. The height of the meniscus was monitored over time in grid and rectilinear capillaries of sizes 1–4 mm (Figure 4a). The data showed higher capillary rise values for the grid samples than for rectilinear samples at all of the examined time points for a = 1 and 1.5 mm. For 2, 3 and 4 mm capillary sizes, the abovementioned trend changes after 5 s, and interestingly the rectilinear configuration produces a higher capillary rise than the grid. This phenomenon can also be observed in Figure 3 in the case of maximum capillary rise. The underlying mechanism is unknown; nevertheless, one can conclude that above a certain capillary size, the non-continuous walls contribute an effect to the capillary rise.

The capillary size effect is also shown in Figure 4. As was expected and discussed earlier, the higher the capillary size, the lower the capillary rise at a certain time point. One of the criteria to design a 3D printed sorbent is that fast sorption and release of oil is facilitated. Thus, the relative capillary rise provides more meaningful information. Figure 4b shows the normalized capillary rise as a function of time for a = 1 and 4 mm for grid and rectilinear configurations when PLA is used. The data indicate that at any given moment, the larger the capillary size, the higher the normalized capillary rise. It can also be noted that the grid configuration further promotes the normalized capillary rise compared to the rectilinear structure; therefore, the oil rises faster when the capillary is constructed by continuous walls in contrast to when it is constructed by non-continuous walls. Hence, it can be concluded that the non-continuous wall promotes the capillary rise; however, it hinders the rate at which the oil gets absorbed compared to the continuous wall.

Another criterion for engineered sorbents is to maximize the amount of absorbed oil in relation to the weight of the sorbent. In rectilinear configuration, the calculated sorption is 0.7 g of oil to 1 g of sorbent, 0.7 g/g, for 1 mm capillary size, and it increases to 2.7 g/g for 4 mm. In practice, this means that the absolute amount of absorbed oil after 10 s rises from 0.58 mg to 2.85 mg for one capillary of 1 mm to 4 mm capillary sizes, respectively, indicating a ~5 times increase in the absorbed oil.

**Figure 5:**
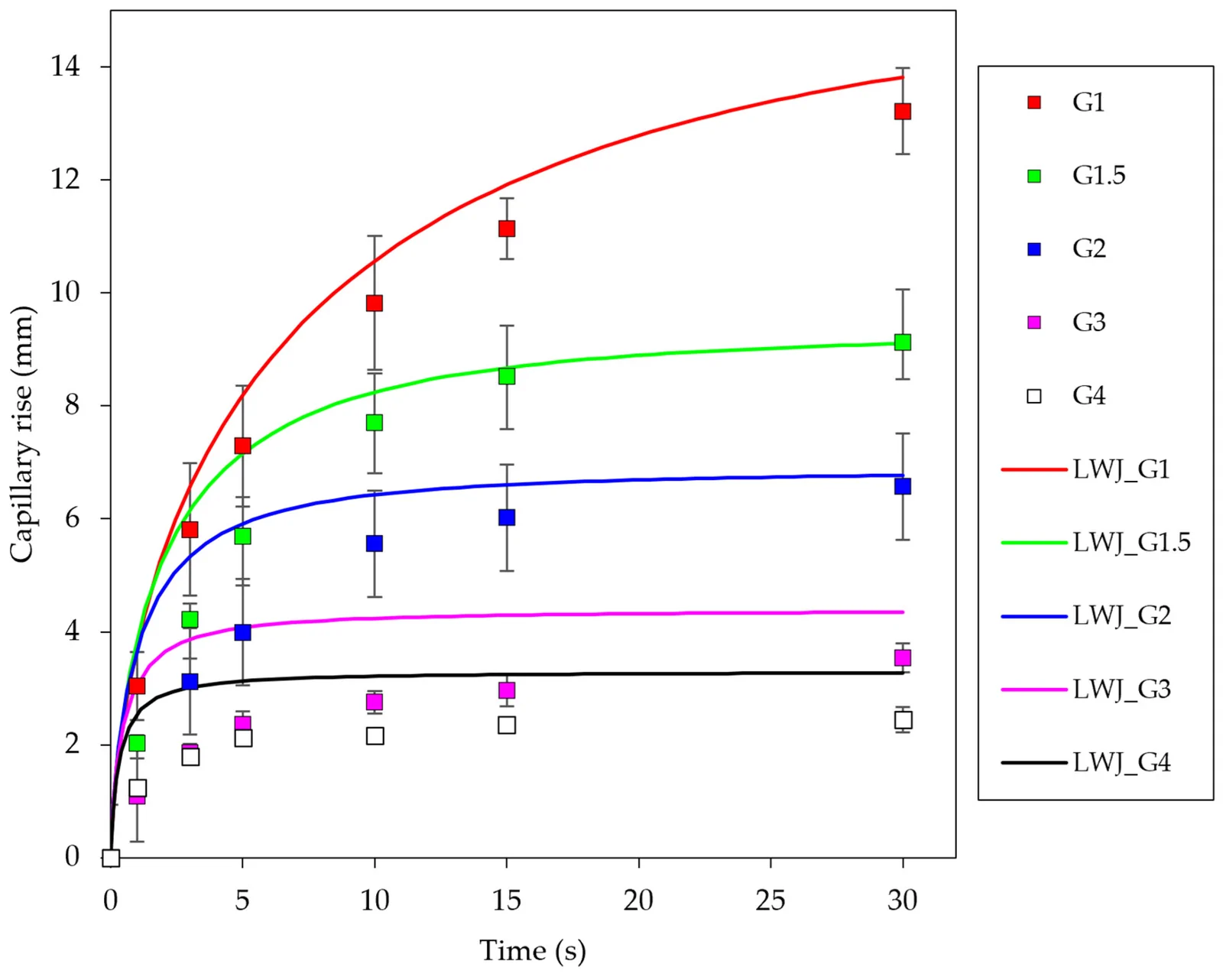
Equation-based and experimental sorption kinetics for PLA and 5W30 motor oil for a = 1, 1.5, 2, 3 and 4 mm in grid configuration.

The flow kinetics in capillary tubes is most commonly described with the Lucas–Washburn equation (LW), based on the research of Lucas and Washburn in capillary flow [34,35]. Originally, the equation was derived for horizontal capillaries when there is perfect wetting. The LW was later implemented for vertical capillaries, as well as expanded with new terms to account for a dynamically changing contact angle [36,37,38,39]. For instance, an equation developed by Joos et al. (LWJ) is based on the Laplace pressure and hydrostatic pressure, law of Poiseuille, and an experimental formula for the dynamic contact angle [39]. Their equation was derived with a 0° static contact angle in mind, which resulted in a simplified formula. In our case, the static OCA is ~30° for the PLA-5W30 oil pairing, so the equation needed to be modified to account for this change. The adjusted equation for the rate of capillary rise is as follows:

(6) $$\frac{dx}{dt}=\frac{\gamma r}{4\eta x}\left(\cos\theta -2\left(1+\cos\theta \right){\left(\frac{\eta }{\gamma }\frac{dx}{dt}\right)}^{\frac{1}{2}}\right)-\frac{\rho gr^{2}}{8\eta }$$

where x is the capillary rise. The complete derivation is presented in the Supplementary Material. The differential equation was numerically integrated in MATLAB Simulink (ver. R2026a) using the ODE45 and built-in loop solvers, with the initial condition of x_0_ = 10^−6^ m. The equation contains the capillary radius r, which was equated to the half of the side length of a square capillary, a/2. The resulting graphs along with the experimental data are shown in Figure 5. The data show a slower sorption in practice than what the model suggests; however, Equation (6) qualitatively describes the capillary effect. The lower sorption is most probably due to the surface roughness of the 3D printed capillaries, since the LWJ equation was developed for capillaries with smooth surfaces.

### 3.3. Viscosity Effect

**Figure 6:**
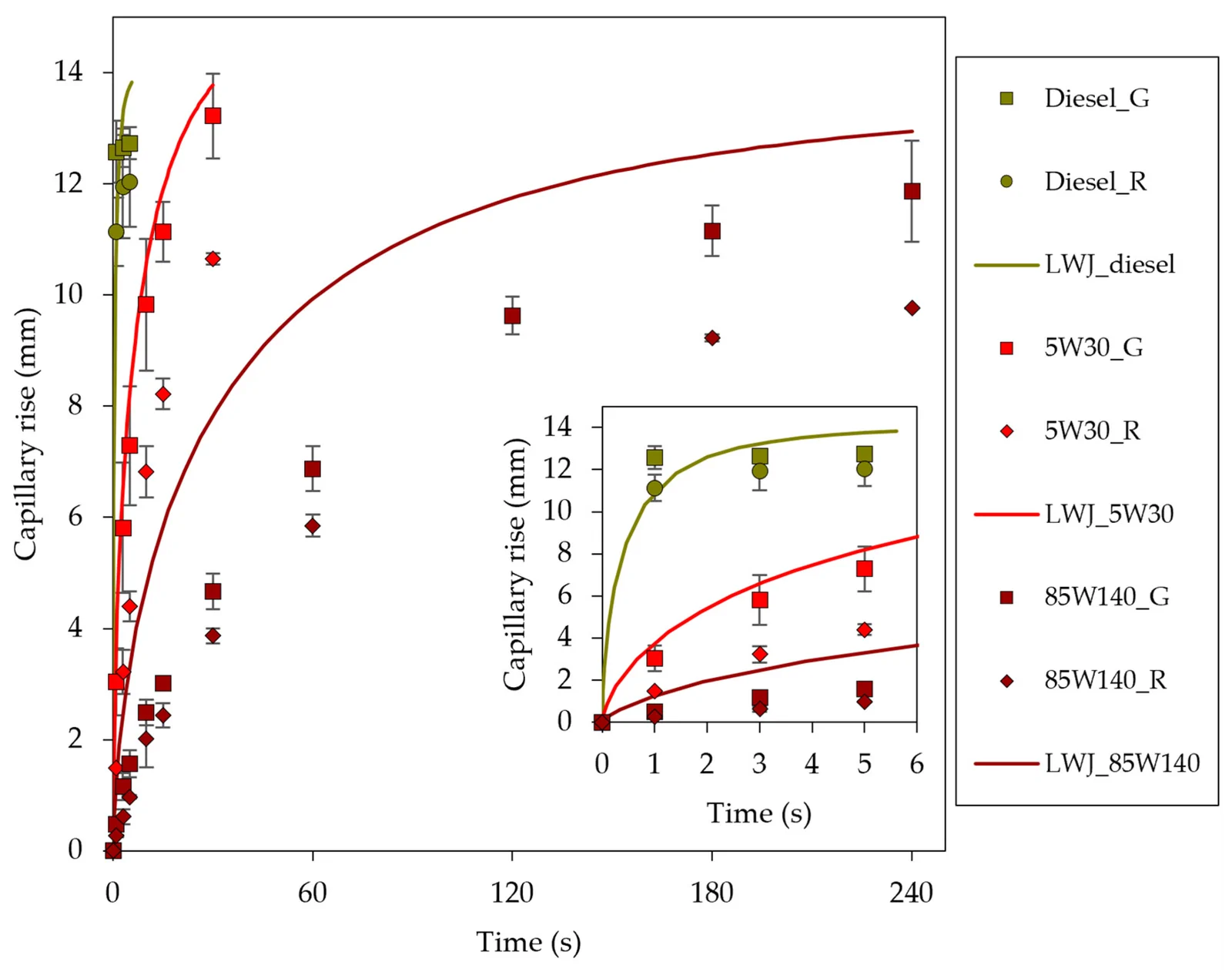
Capillary rise as a function of time for PLA with diesel, 5W30 and 85W140 oils, a = 1 mm.

In addition to the 5W30 motor oil, 1 mm PLA capillaries were also tested with diesel and 85W140 gear oil. The biggest difference in this case was the viscosity of the oils. Compared to the 5W30 with a dynamic viscosity of ~126.6 mPa·s, the viscosity of diesel was lower by an order of magnitude at 8.6 mPa·s, while the viscosity of 85W140 oil was 725 mPa·s. In the Lucas–Washburn equation, the rate of capillary rise is inversely proportional to dynamic viscosity. Figure 6 shows that the diesel sorption in 1 mm PLA capillaries was significantly faster than the motor oil, reaching maximum height in 3 s, while it took about 4 min for the transmission oil to reach the maximum height. The small differences in maximum capillary rise are due to the differences in surface tension and density of the oils. The plots generated with the LWJ equation show an increasing deviation from the experimental data as the viscosity increased.

### 3.4. Sorbent Material Effect

**Figure 7:**
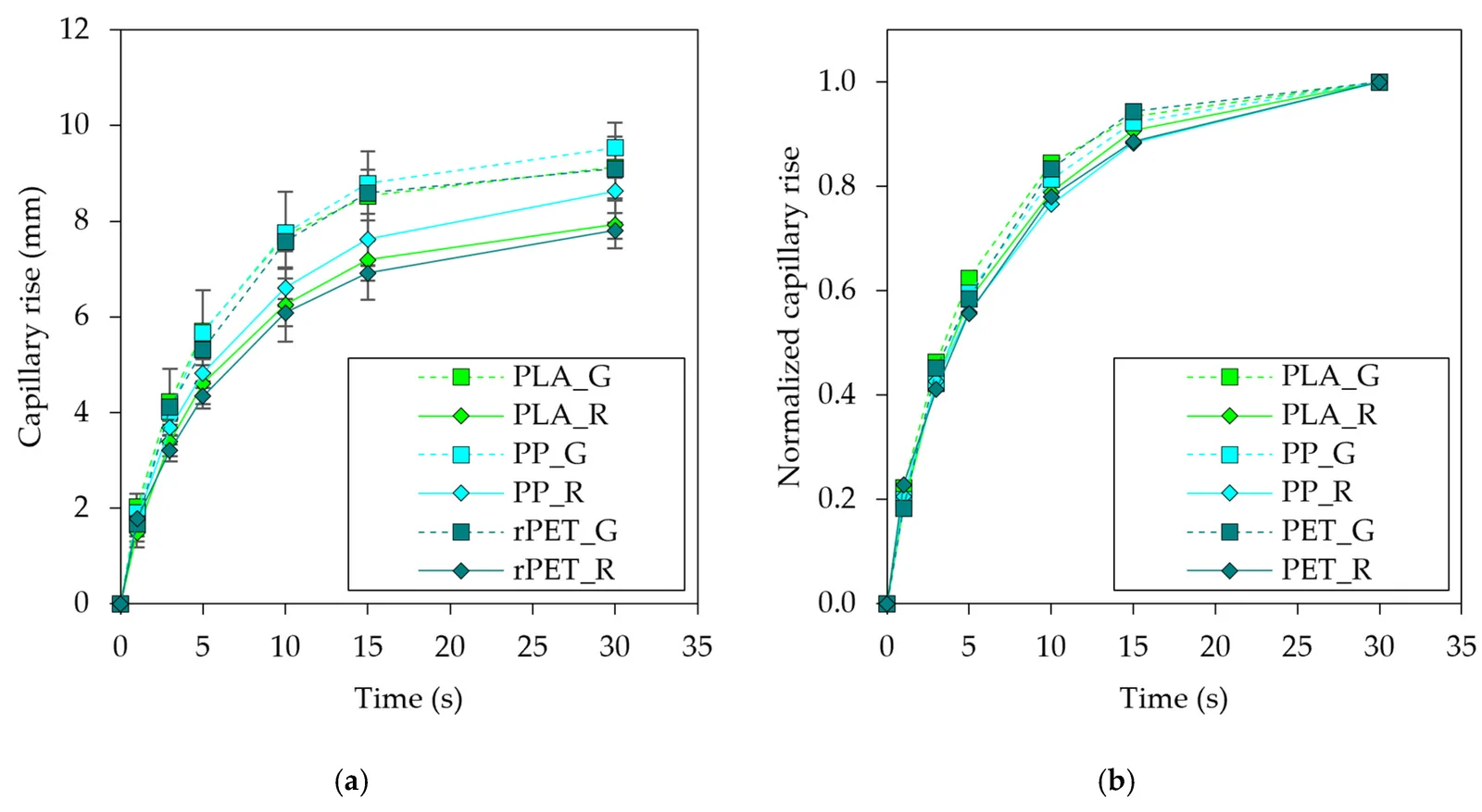
(**a**) Sorption kinetics for 5W30 oils with PLA, rPET and PP sorbents, a = 1.5 mm. (**b**) Normalized capillary rise.

The 5W30 motor oil was also tested with 1.5 mm capillary size for PLA, rPET and PP capillaries to investigate the effect of sorbent material affinity on sorption kinetics and maximum capillary rise. In each case, the grid structures reached higher capillary rise than their rectilinear counterparts. The PLA and rPET grid capillaries performed almost identically, 9.13 ± 0.65 mm and 9.1 ± 0.19 mm at 30 s, respectively, while PP showed a higher capillary rise than the other two with 9.53 ± 0.53 mm. The same trend is seen with the rectilinear capillaries but with the difference more pronounced. The measured capillary sizes were 1.45 ± 0.01 and 1.53 ± 0.03 mm for PLA grid and rectilinear, 1.48 ± 0.05 and 1.55 ± 0.02 for rPET, and 1.43 ± 0.05 and 1.49 ± 0.05 mm for PP, respectively. In each case, the rectilinear capillaries were larger than the grid counterparts, and the rPET was the largest, followed by the PLA, and finally the PP. The values align with the observed trend, as larger capillaries lead to lower maximum capillary rise and can partially explain the results. Another factor is the affinity of the materials to the oil. As seen in Table 2, PLA had the highest OCA at 29.7 ± 6.1, then the PP at 21.3 ± 1.2, and the rPET at 17.6 ± 3, which further increases the gap between PLA and PP, while causing rPET catch up to PLA in terms of maximum capillary rise. Figure 7b shows the normalized capillary rise for each material. As seen before, the grid configuration showed a faster relative sorption. On the other hand, the grid and rectilinear graphs respectively overlap, indicating that the material of the sorbent has little to no effect on the kinetics.

### 3.5. Oil–Water Separation

**Figure 8:**
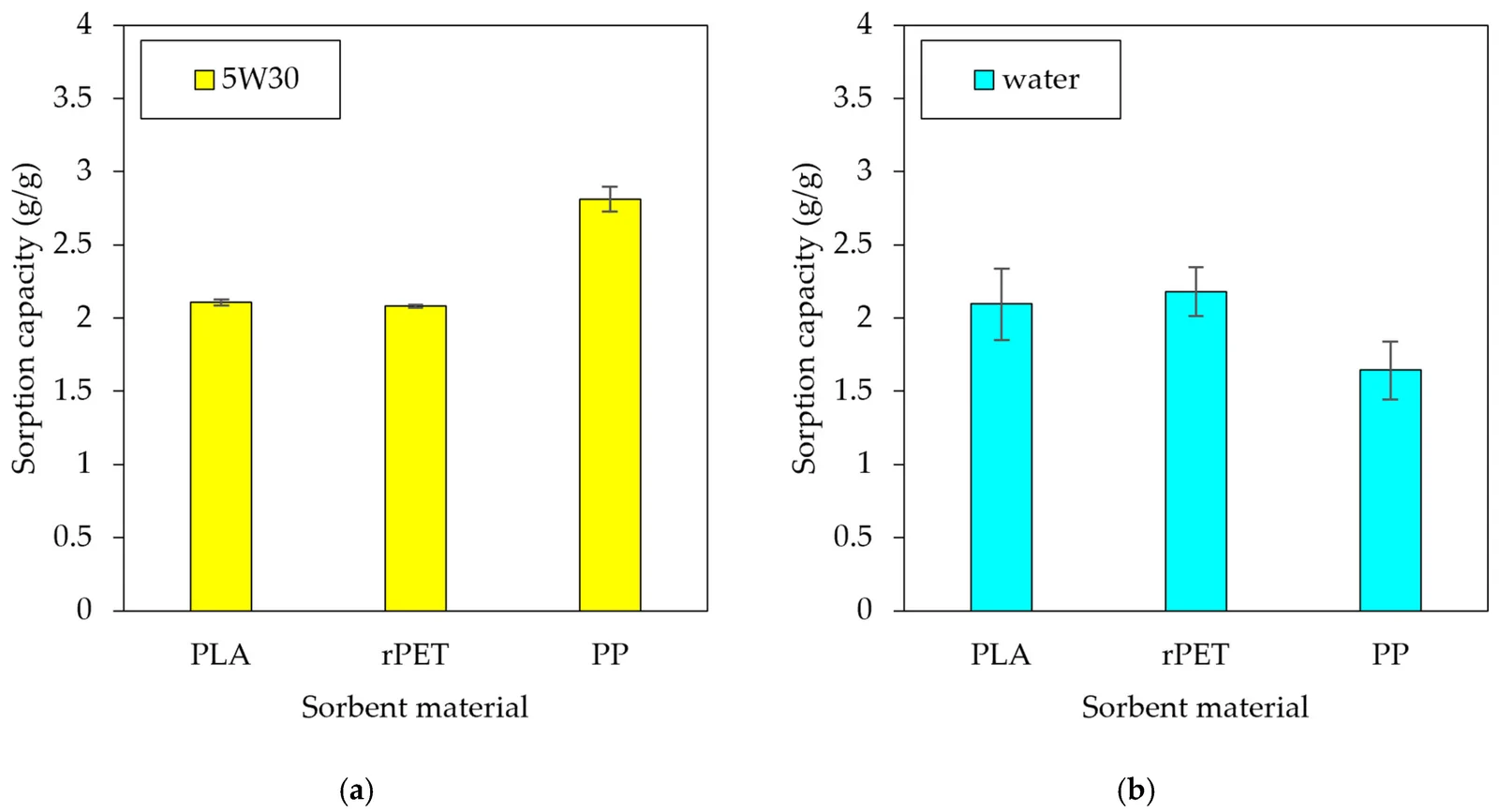
Sorption characteristics of 1.5 mm PLA, rPET and PP sorbents for (**a**) 5W30 oil and (**b**) water.

To examine the usability of the printed structures as sorbents, oil sorption experiments were carried out with rectilinear specimens of 5 mm thickness and 1.5 mm capillary size using 5W30 oil. All specimens became fully saturated and did not release any oil upon removal from the reservoir. The PLA and rPET filaments have almost identical densities at 1.27 and 1.29 g/cm^3^, respectively, and the results show that the sorption capacity was also identical at 2.1 g/g (Figure 8a). The PP specimens showed a higher 2.8 g/g sorption capacity with the same volume, which is due to the lower density of PP, 0.9 g/cm^3^.

Placing the specimens into pure water caused it to penetrate the porous structure. Both PLA and rPET fully submerged, and the rPET specimens sunk to the bottom, while the PP specimens remained halfway above the water surface. The data in Figure 8b also show that PP had significantly lower water uptake than the other two, even with its lower density. The ~2.1 g/g water uptake of the PLA and rPET specimens corresponds to 1.19 g, whereas it was 1.6 g/g in the case of PP is 0.67 g, meaning that in absolute terms the water uptake for the PP specimen was less than half of that of PLA and rPET.

**Figure 9:**
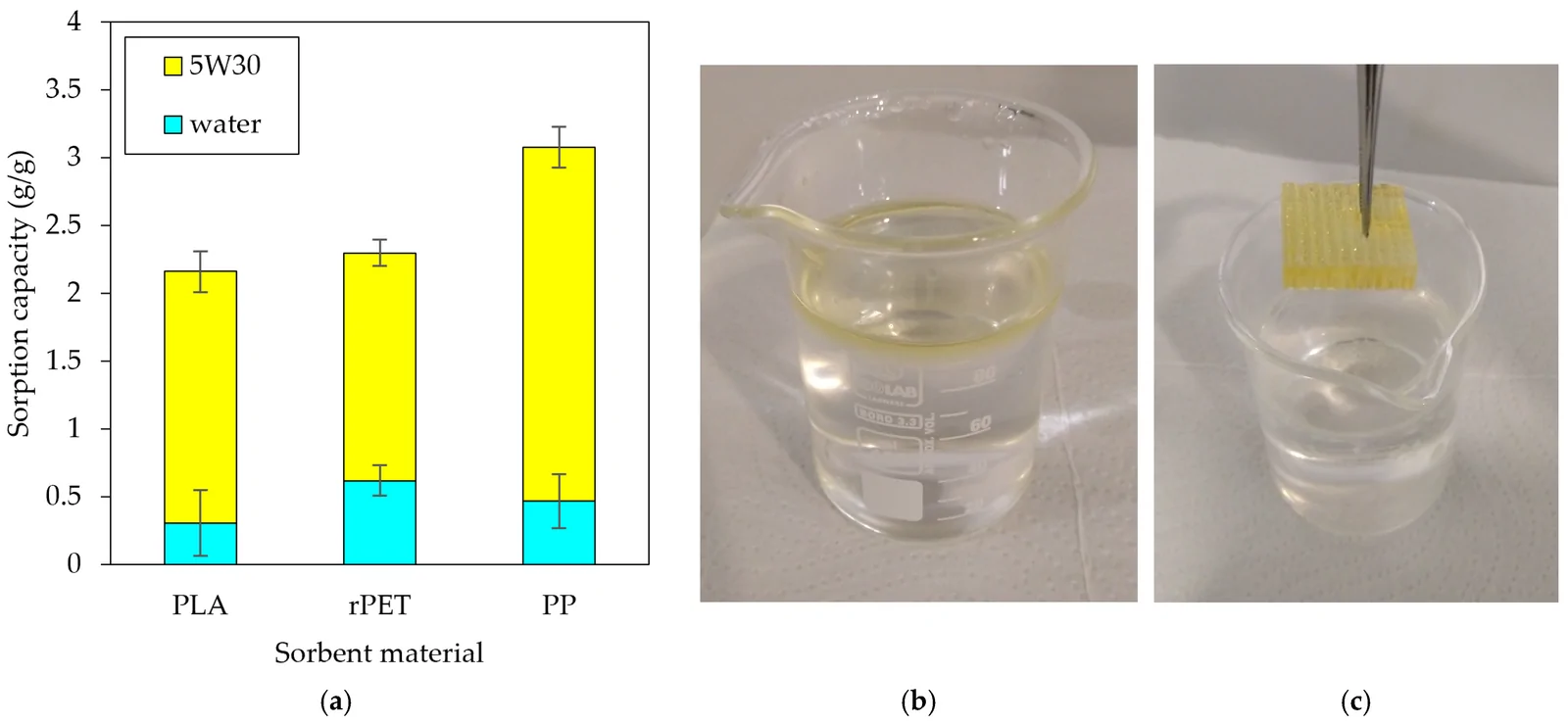
Oil–water separation experiments results: (**a**) sorption capacity of oil and water for PLA, rPET and PP, a = 1.5 mm; (**b**) medium before sorption; and (**c**) specimen with the collected oil.

The oil–water separation experiment results can be seen in Figure 9a. Specimens of all three materials collected a significant amount of water along with the oil. On average, PLA collected oil and water in a ratio of 86/14 and 86.4% of the added oil. These numbers are 73/27 and 75.4% for rPET, respectively, and 85/15 and 79.5% for PP, respectively. Overall, the oil–water separation performance of the three materials is similar, with PP having the largest oil uptake on a g/g basis, while rPET collected the most water. 3D printed structures are not unique in this aspect, as microfibrous mats that had WCA > 125°, thus are considered hydrophobic, also collected a significant amount of water during oil–water separation experiments when the amount of oil was less than what would have saturated the samples [40]. Figure 9b,c show the oil layer on top of the water and a PLA sample after oil collection, with most of the oil removed from the water surface.

### 3.6. Sorption Engineering

PLA was chosen as model material to engineer a sorbent over PP, although PP has a higher oil sorption capacity. 3D printing of PP sorbents poses some technical difficulties, showing a high degree of thermal warping and insufficient bed adhesion, whereas PLA has exceptional printability that is well documented in the literature. Furthermore, rectilinear configuration was selected due to its higher porosity.

**Figure 10:**
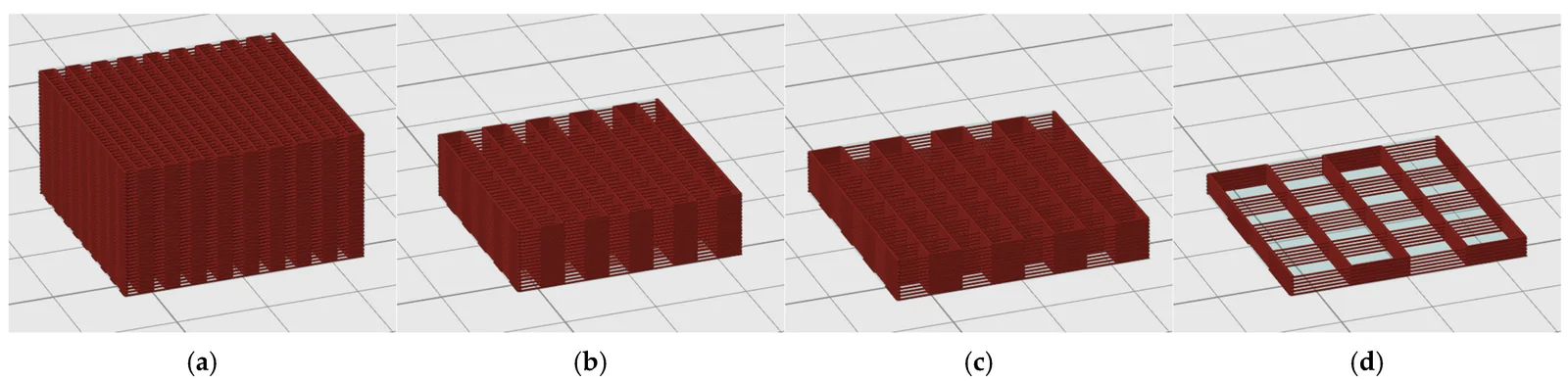
3D printed rectilinear infills with (**a**) 1 mm, (**b**) 2 mm, (**c**) 3 mm, (**d**) 6 mm capillary sizes and their respective maximum active heights.

When working with fiber mats, the dimensions of the mats are usually not discussed, and instead mass and porosity are the only quantities that are considered during an experiment. The fiber mats are often sheets with negligible thickness, and the pore size is in the 1–10 micrometer range, so the capillary height is usually ignored. In our case, the thickness of the specimens is important, because above the maximum capillary rise any additional height is extra material that cannot be used for sorption. Figure 10 shows the 1, 2, 3, and 6 mm rectilinear infill-based capillaries with their respective maximum useful thicknesses of 14.4, 7.2, 4.8, and 2.4 mm, respectively, acquired with Equation (5).

**Figure 11:**
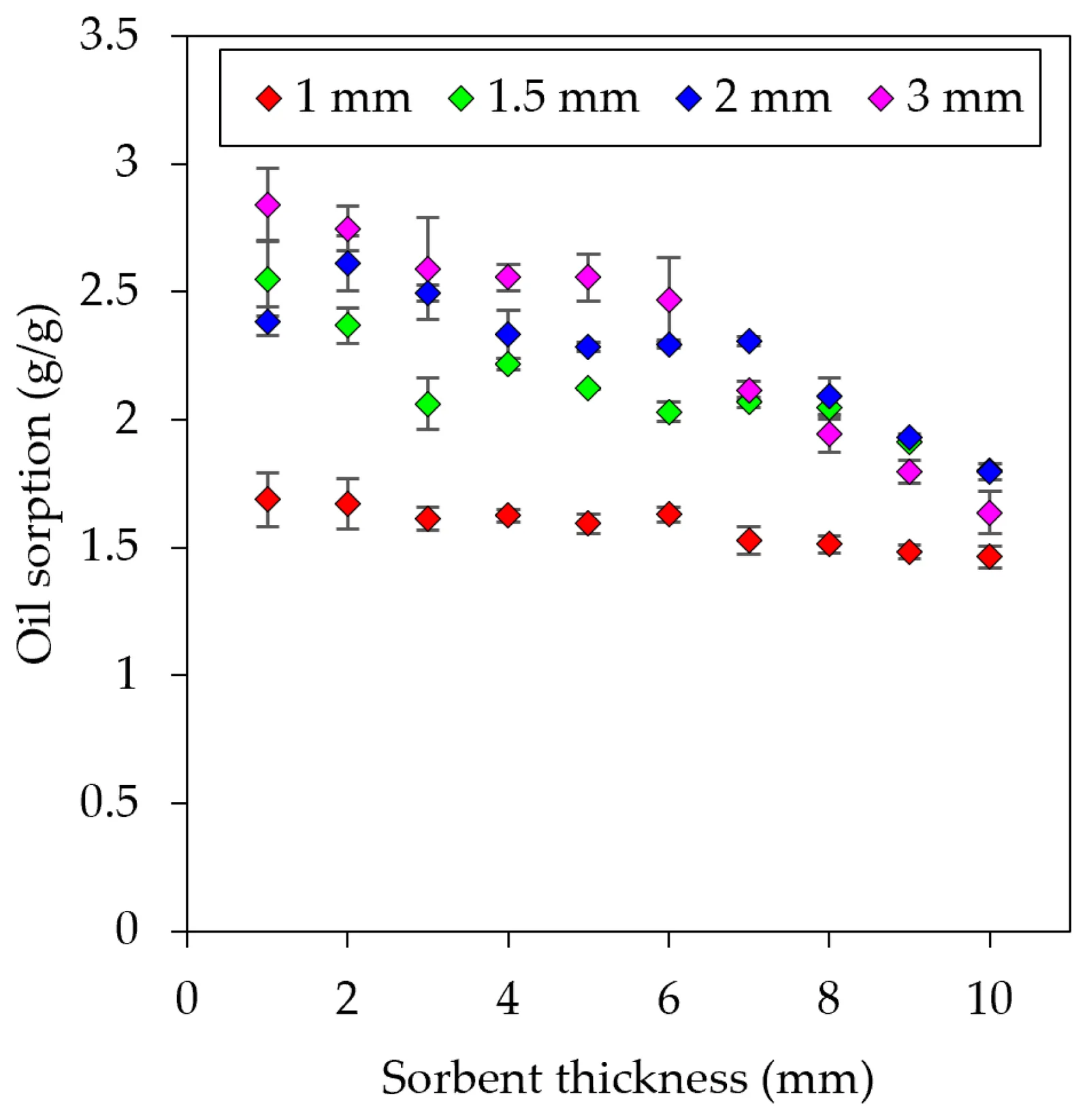
Sorption capacities of rectilinear specimens as a function of sorbent thickness and capillary size.

The oil sorption experiments with 1, 1.5, 2, and 3 mm rectilinear capillaries of the same area of 25 × 25 mm^2^ and varying thicknesses can be seen in Figure 11. Specimens with 4, 5 and 6 mm capillary sizes could not maintain stable menisci upon being removed from the oil, resulting in uncontrolled oil leakage. At low sorbent thickness (<7 mm), as the capillary size increases, the sorption capacity also increases because all the specimens can saturate with oil, but the large capillary specimens have less material in them due to higher porosity. There is a point for each capillary size where the sorption capacity starts to decrease, and the larger the capillary, the sooner this happens, with 8, 7 and 6 mm sorbent thicknesses for 1.5, 2 and 3 mm capillary sizes, respectively. This is due to reaching the maximum capillary rise for the given capillary size, and any additional height only provides the benefit of extra surface area that can be covered with oil but cannot retain it. In addition, the measured sorption capacity values were lower than the predicted values based on the model at larger capillary sizes, with 2 mm 2.61 vs. 2.9 g/g and 3 mm 2.84 vs. 4.2 g/g, whereas in the case of smaller capillary sizes they were similar: 1 mm 1.67 vs. 1.6 g/g and 1.5 mm 2.55 vs. 2.5 g/g.

**Figure 12:**
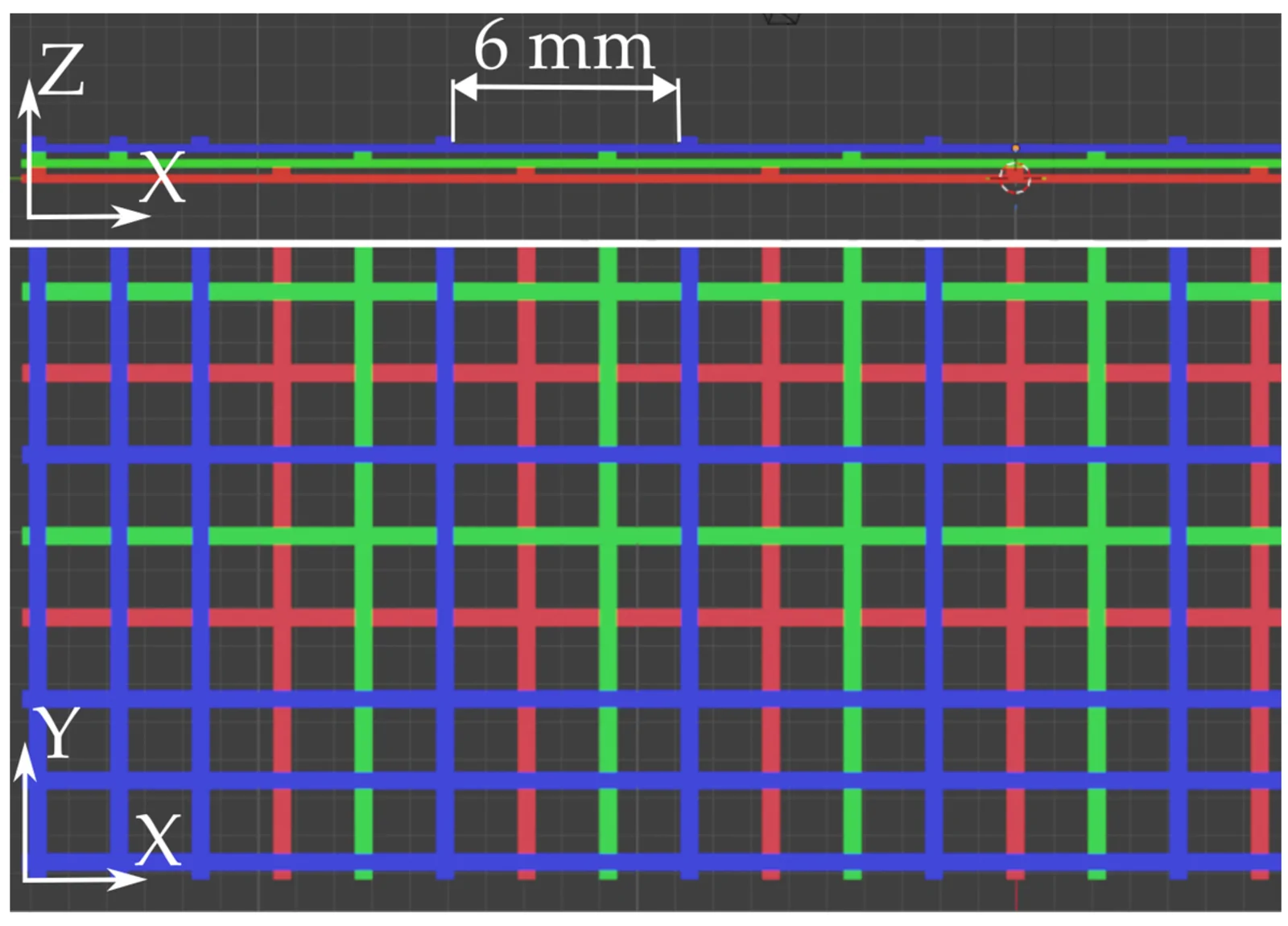
3D model in Blender of engineered sorbent with shifted 6 mm capillaries: red—bottom double layer, green—middle double layer, and blue—top double layer.

Based on the measurements, it can be stated that the capillary size at which the specimens still hold the oil and can have a high porosity at reasonable thickness are the 2 and 3 mm capillaries. As seen previously, these specimens can hold 2.6–2.8 g/g oil, as long as the thickness of the sorbents is lower than 6 mm. A way to maintain the de facto capillary size of 2 mm but use less material in the production of the specimens can be achieved by shifting the layers of a larger sample by a certain amount. Every two layers of a 6 mm rectilinear mesh were shifted by 2 mm both in the X and Y directions, technically creating a sorbent with a = 2 mm, if viewed from above, but without a continuous capillary wall (Figure 12). This solution provides a sorbent that has a capillary size of 2 mm, yet its porosity resembles that of specimens with 6 mm capillary size, 93%.

**Figure 13:**
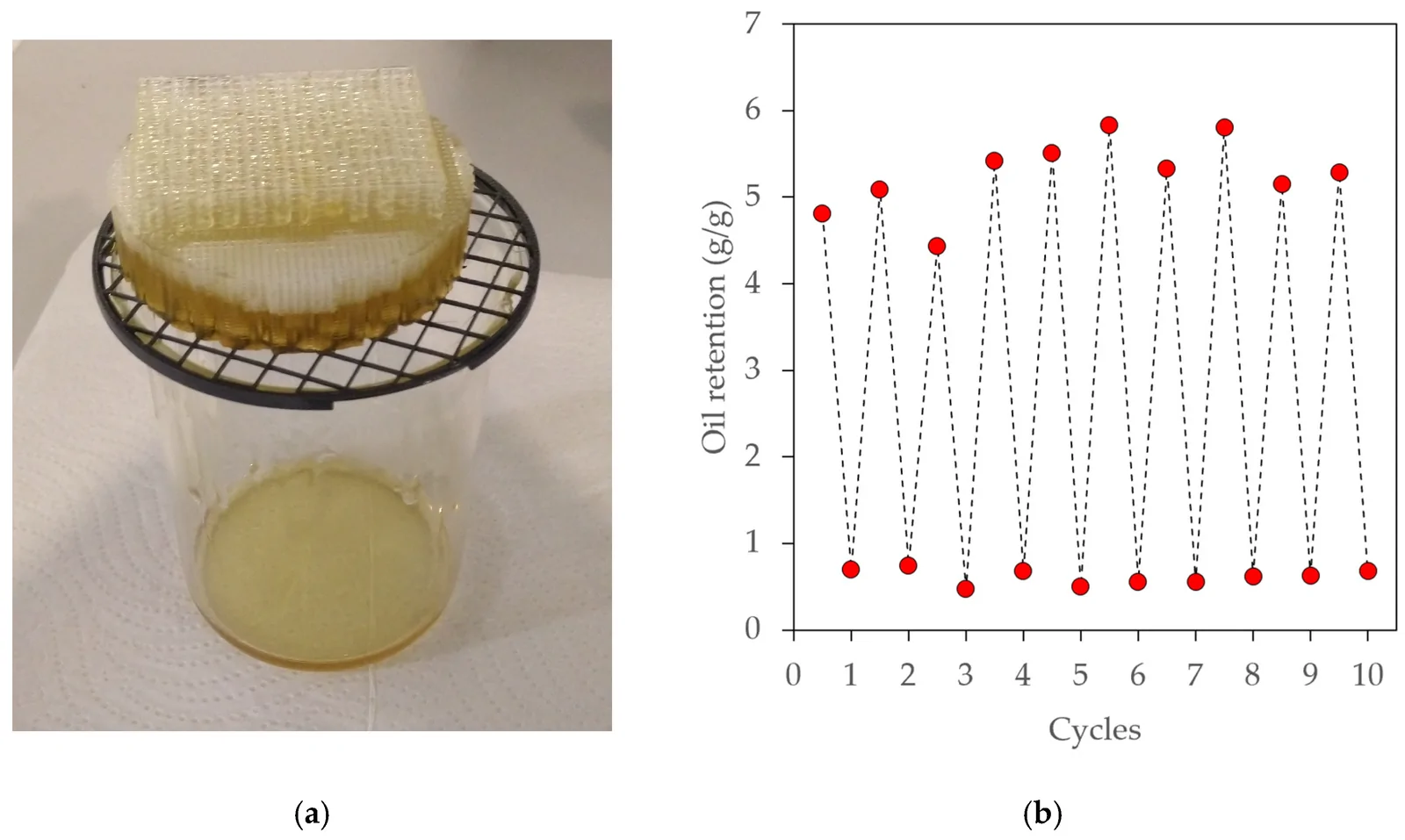
Oil sorption with the engineered sorbent. (**a**) Drainage setup and (**b**) reusability represented by the oil retention over 10 sorption–release cycles.

The sorption with the engineered specimen took 10 s to complete, after which it was lifted from the oil bath. During the 30 s drain time, 2–3 drops of oil got released, but otherwise the sorbent retained the oil. The oil was extracted from the sorbent by placing it on a cylindrical pad containing 1 mm capillaries (Figure 13a). It is important to underline that the drainage does not require any specialized equipment as it is governed by gravity. The draining process took 7–8 s. The sorption–release cycle was repeated 10 times (Figure 13b). After draining, the leftover oil in the specimen was 0.42 ± 0.09 g/g, mainly consisting of the oil that was wetting the surface of the sorbent. The measured sorption capacity was 5.26 ± 0.61 g/g, falling short of the 9 g/g predicted value; nonetheless, it practically doubled that of the standard 2 mm rectilinear structure. The specimen had dimensions of 40 × 52.5 × 7.2 mm and weighed 1.45 g, and so 5.26 g/g oil equals 7.63 g in absolute terms.

## 4. Conclusions

Fiber-based sorbents were successfully prepared by FFF 3D printing. To study the sorption process, we investigated the effects of sorbent structure (grid and rectilinear), capillary size, oil viscosity and sorbent material. Although the grid structure provided marginally faster sorption kinetics, the rectilinear configuration was desirable due to the higher porosity. Capillary size has a dramatic effect on the sorption capacity, and so does the viscosity of the absorbable liquid. The material of the sorbent did not show a significant effect when comparing PLA, rPET and PP. The sorption kinetics were also investigated, and a modified Lucas–Washburn equation was used to model the phenomenon. The data suggest that the equation describes the process only qualitatively. All sorbents, regardless of the used material, collected more than 75% of the oil during oil–water separation experiments. A sorbent structure was proposed that further increased the porosity, thus increasing the sorption capacity to 5.26 g/g. The reusability of fiber-based sorbents presented in the literature is either not discussed, or it requires special equipment such as a centrifuge or mechanical press. In our case, the oil release is driven by gravity and the drain time was fast, under 8 s. The sorption capacity of the 3D printed sorbents is lower than that reported for nanofibers prepared by electrospinning or centrifugal spinning. However, if the applicability in real-life oil sorption is considered, the 3D printed sorbents are a promising alternative to nanofibers due to the easy and fast reusability process and their inexpensive production.

## Figures and Tables

**Table 1:** Characteristic properties of the used oils.

Liquid	γ (mN/m)	*ρ* (g/cm^3^)	η (mPa·s)
diesel	26.58 ± 0.06	0.835	8.6 @ 7.25 1/s shear rate
5W30	31.48 ± 0.05	0.860	128.7 @ 7.25 1/s shear rate
85W140	33.02 ± 0.01	0.903	725 @ 2.89 1/s shear rate

**Table 2:** WCA and OCA data of the investigated oils and polymeric surfaces.

	PLA	rPET	PP
WCA (°)	81.46 ± 1.23	86.12 ± 1.42	90.64 ± 5.37
OCA (°) of diesel	24.68 ± 3.24	--	--
OCA (°) of 5W30	29.72 ± 6.08	17.58 ± 2.98	21.28 ± 1.29
OCA (°) of 85W140	39.02 ± 6.84	--	--

**Table 3:** Theoretical and measured capillary sizes of PLA specimens.

a_theo_ (mm)	Grid		Rectilinear	
	a_meas_ (mm) *	Error	a_meas_ (mm) *	Error
1	0.83 ± 0.03	−17.5%	1.03 ± 0.06	2.8%
1.5	1.37 ± 0.02	−8.4%	1.52 ± 0.05	1.1%
2	1.87 ± 0.04	−6.4%	2.08 ± 0.05	4.1%
3	2.94 ± 0.02	−2.0%	3.05 ± 0.09	1.8%
4	3.92 ± 0.04	−1.9%	3.98 ± 0.01	−0.5%

* Six samples were measured.

## Data Availability

Data are contained within the article.

## Supplementary Materials

The following supporting information can be downloaded at: https://www.mdpi.com/article/10.3390/polym18172138/s1.

## Abbreviations

The following abbreviations are used in this manuscript:

CA	contact angle
FFF	fused filament fabrication
LW	Lucas–Washburn equation
LWJ	Lucas–Washburn–Joos equation
OCA	oil contact angle
PLA	polylactic acid
PP	polypropylene
PS	polystyrene
rPET	recycled polyethylene terephthalate
WCA	water contact angle
