# Supplementary material for: Oil Sorption and Oil–Water Separation Using 3D Printed Sorbents Manufactured with Fused Filament Fabrication
Source: Polymers (Basel). 2026 Sep 2;18(17):2138. doi: 10.3390/polym18172138 (PMC13568339; doi:10.3390/polym18172138)
Supplement: Supplementary file 1 [file polymers-18-02138-s001.zip › polymers-4512865-supplementary.pdf]

Supplementary Material

Oil sorption and oil-water separation using 3D printed sorbents manufactured with Fused Filament Fabrication

József Kántor [1] and Attila-Levente Gergely [1,*]

[1] Department of Mechanical Engineering, Faculty of Technical and Human Sciences, Sapientia Hungarian University of Transylvania, Târgu Mureş, Târgu Mureş/Corunca, Calea Sighișoarei nr. 2., 540485 Târgu Mureş, Romania; kantorjozsef@ms.sapientia.ro (J.K.), agergely@ms.sapientia.ro (A.L.G.)

Derivation of the capillary rise equation

The steps of the derivation were taken from Joos et al., reference [39] in the article. The only difference is that they assumed perfect wetting on the capillary walls (θ = 0, cos(θ) = 1), while in our case θ in non-zero, so the dynamic contact angle, θ_d is kept in its original form throughout the derivation.

The pressure difference at the open surface level inside the vertical capillary compared to the atmospheric pressure is given by the difference of the Laplace pressure and the hydrostatic pressure of the liquid column:

$$\Delta p = \frac{2\gamma \cos \theta_d}{r} - \rho g x \quad \text{(S1)}$$

The dynamic contact angle based on an empirical formula is as follows:

$$\theta_d = \cos \theta - 2(1 + \cos \theta) \left(\frac{\eta}{\gamma} \frac{dx}{dt}\right)^{\frac{1}{2}} \quad \text{(S2)}$$

The volumetric flow rate in the capillary can be calculated either from the law of Poiseuille or the moving meniscus with a certain velocity.

$$\mathrm{f} = \frac{\pi r^4}{8\eta x} \Delta p = \pi r^2 v \quad \text{(S3)}$$

Rearranging Eq. S3 to get the rate of capillary rise:

$$v = \frac{dx}{dt} = \frac{r^2}{8\eta x} \Delta p \quad \text{(S4)}$$

Substituting Eq. S2 into S1 yields the following:

$$\Delta p = \frac{2\gamma}{r}\left(\cos\theta - 2(1+\cos\theta)\left(\frac{\eta}{\gamma}\frac{dx}{dt}\right)^{\frac{1}{2}}\right) - \rho g x \tag{S5}$$

Substituting Eq. S5 into S4 gives the final formula for the rate of capillary rise:

$$\frac{dx}{dt} = \frac{\gamma r}{4\eta x}\left(\cos\theta - 2(1+\cos\theta)\left(\frac{\eta}{\gamma}\frac{dx}{dt}\right)^{\frac{1}{2}}\right) - \frac{\rho g r^2}{8\eta} \tag{S6}$$

The differential equation is not in an explicit form, evidenced by the presence of dx/dt on both sides of the equation. MATLAB Simulink has a solver that can handle loops, and it can provide a solution for the equation in its current form.
